# Supplementary material for: Resolving the full spectrum of human genome variation using Linked-Reads
Source: Genome Res. 2019 Apr;29(4):635–45. doi: 10.1101/gr.234443.118 (PMC6442396; doi:10.1101/gr.234443.118)
Supplement: Supplemental Material [file supp_gr.234443.118_Supplemental_longranger-2.2.zip › longranger-2.2.tar.bz2!/lr2.2-public/lib/stan/cmdstan/stan/doc/api/html/structstan_1_1io_1_1stan__csv__metadata.html]

Stan: stan::io::stan\_csv\_metadata Struct Reference


|  |  |
| --- | --- |
|  | Stan  2.5.0  probability, sampling & optimization |


- Main Page
- Related Pages
- Namespaces
- Classes
- Files

- Class List
- Class Index
- Class Hierarchy
- Class Members

All Classes Namespaces Files Functions Variables Typedefs Enumerations Enumerator Friends Macros Pages

- stan
- io
- stan\_csv\_metadata

Public Member Functions |
Public Attributes |
List of all members

stan::io::stan\_csv\_metadata Struct Reference

`#include <stan_csv_reader.hpp>`

|  |  |
| --- | --- |
| Public Member Functions | |
|  | stan\_csv\_metadata () |
|  | |

|  |  |
| --- | --- |
| Public Attributes | |
| int | stan\_version\_major |
|  | |
| int | stan\_version\_minor |
|  | |
| int | stan\_version\_patch |
|  | |
| std::string | model |
|  | |
| std::string | data |
|  | |
| std::string | init |
|  | |
| size\_t | chain\_id |
|  | |
| size\_t | seed |
|  | |
| bool | random\_seed |
|  | |
| size\_t | num\_samples |
|  | |
| size\_t | num\_warmup |
|  | |
| bool | save\_warmup |
|  | |
| size\_t | thin |
|  | |
| bool | append\_samples |
|  | |
| std::string | algorithm |
|  | |
| std::string | engine |
|  | |

## Detailed Description

Definition at line 16 of file stan\_csv\_reader.hpp.

## Constructor & Destructor Documentation

|  |  |  |  |  |
| --- | --- | --- | --- | --- |
| stan::io::stan\_csv\_metadata::stan\_csv\_metadata | ( |  | ) |  |

inline

Definition at line 35 of file stan\_csv\_reader.hpp.

## Member Data Documentation

std::string stan::io::stan\_csv\_metadata::algorithm

Definition at line 32 of file stan\_csv\_reader.hpp.

bool stan::io::stan\_csv\_metadata::append\_samples

Definition at line 31 of file stan\_csv\_reader.hpp.

size\_t stan::io::stan\_csv\_metadata::chain\_id

Definition at line 24 of file stan\_csv\_reader.hpp.

std::string stan::io::stan\_csv\_metadata::data

Definition at line 22 of file stan\_csv\_reader.hpp.

std::string stan::io::stan\_csv\_metadata::engine

Definition at line 33 of file stan\_csv\_reader.hpp.

std::string stan::io::stan\_csv\_metadata::init

Definition at line 23 of file stan\_csv\_reader.hpp.

std::string stan::io::stan\_csv\_metadata::model

Definition at line 21 of file stan\_csv\_reader.hpp.

size\_t stan::io::stan\_csv\_metadata::num\_samples

Definition at line 27 of file stan\_csv\_reader.hpp.

size\_t stan::io::stan\_csv\_metadata::num\_warmup

Definition at line 28 of file stan\_csv\_reader.hpp.

bool stan::io::stan\_csv\_metadata::random\_seed

Definition at line 26 of file stan\_csv\_reader.hpp.

bool stan::io::stan\_csv\_metadata::save\_warmup

Definition at line 29 of file stan\_csv\_reader.hpp.

size\_t stan::io::stan\_csv\_metadata::seed

Definition at line 25 of file stan\_csv\_reader.hpp.

int stan::io::stan\_csv\_metadata::stan\_version\_major

Definition at line 17 of file stan\_csv\_reader.hpp.

int stan::io::stan\_csv\_metadata::stan\_version\_minor

Definition at line 18 of file stan\_csv\_reader.hpp.

int stan::io::stan\_csv\_metadata::stan\_version\_patch

Definition at line 19 of file stan\_csv\_reader.hpp.

size\_t stan::io::stan\_csv\_metadata::thin

Definition at line 30 of file stan\_csv\_reader.hpp.

---

The documentation for this struct was generated from the following file:

- src/stan/io/stan\_csv\_reader.hpp


---

[ Stan Home Page ]

*© 2011–2014,
Stan Development Team.*
